# Philippine Mental Health Act: Just an Act? A Call to Look Into the Bi-directionality of Mental Health and Economy

**DOI:** 10.3389/fpsyg.2021.706483

**Published:** 2021-07-21

**Authors:** Nicholle Mae Amor Tan Maravilla, Myles Joshua Toledo Tan

**Affiliations:** ^1^College of Medicine, Cebu Doctors' University, Mandaue, Philippines; ^2^Department of Natural Sciences, University of St. La Salle, Bacolod, Philippines; ^3^Department of Chemical Engineering, University of St. La Salle, Bacolod, Philippines

**Keywords:** Philippines, mental health act, mental health, happiness and well-being, Northwestern Europe, mental health economics, economy, psychology

## Introduction

It may seem intuitive to say that a healthy economy makes people happy, but little is said about the converse of this statement. Perhaps, we should look into how happy people make an economy healthy. The nature of happiness has been debated on by philosophers for thousands of years, but a benchmark for it that has been accepted by many social and behavioral scientists in the past few decades has been Diener's Subjective Well-being (SWB) (Diener, 2009). Studies that employ the use of SWB show that individuals who report high levels of it tend to live longer with healthier lives, have healthier social relationships, and work more productively (Montagnoli, 2019). Hence, higher levels of SWB could mean good mental health among individuals. Poli et al. (2020) described good mental health as “a state of well-being that allows individuals to cope with the normal stresses of life and function productively.” However, the definition of *good mental health* may vary among cultures, values and traditions. Culture affects how people manifest symptoms, express these symptoms, deal with psychological problems, and decide whether to seek care (Eshun and Gurung, 2009). Because of these differences, the best way to enrich our understanding of mental health might be to evaluate the different perspectives of mentally healthy populations toward it (Vaillant, 2003). Further understanding of mental health is significant because it greatly affects the economy as economies appear to crucially depend on the population's mental health. And so, good mental health is significant for economic growth. According to Knapp and Wong (2020), the economy has a bi-directional relationship with mental health. Economic decline may lead to a greater likelihood of mental-illness due to exposure to risk factors such as social exclusion, poor education, treatment costs, unemployment, and poverty. Mental health problems may also lead to a significant decline in economic activity that results from productivity losses and limited resources for treatment. Thus, the study of economics, particularly mental health economics (MHE), is significant in identifying ways to improve mental health and mental healthcare production and consumption. Unfortunately, to the best of our knowledge and understanding, there are currently no existing studies written on Philippine MHE. And so to help address the economic and mental health crises in the Philippines, we hope to spark discussions that will promote the study of MHE for the good of all Filipinos.

## The State Of Mental Health in the Philippines

The Philippines is an archipelago of over 7000 islands, with over 120 languages, and numerous religions. The country has been occupied and colonized by many foreign powers since 1545 and it only gained full independence in 1946. This colonial history has contributed to the unique Filipino culture, yet the country remains poorly understood because of its late independence and of it being one of only two Christian-majority countries in the Far East (Lally et al., 2019). The cultural beliefs of Filipinos vary in almost every respect. One of the popular beliefs is that depression and anxiety are non-existent, and that mental illnesses are something to be ashamed of. A qualitative study conducted by Tanaka et al. (2018) showed that this stigma is considered to be an effect of the public belief about mental disorders which consist of three themes: First is *familial problems*, wherein the family rejects or disowns the family member who suffers from a mental disorder because they believe that it can be inherited. Second is *unrealistic pessimism and optimism* about the severity of the disorder, wherein the mentally ill either would certainly suffer from severe functional impairment or would be able to overcome any psychological suffering by themselves. Last is the *oversimplified chronic course*, wherein people without mental illnesses apply an acute illness model to those ill, and expect full recovery in the short term.

Because of this stigma, mental health has been given very little attention by the Philippine government and public sectors. Even after the country has recently passed its first Mental Health Act and Universal Health Care Law, only 5% of the healthcare expenditure is directed toward mental health. Also, there are only 7.76 hospital beds and 0.41 psychiatrists per 100,000 people (World Health Organization—Assessment Instrument for Mental Health Systems, 2007; Department of Health, 2018). This ratio was known to be lower than other Western Pacific countries with similar economic statuses, like Malaysia and Indonesia (Lally et al., 2019). The Philippine government does not even provide economic support for organizations that have been involved in the formulation and implementation of mental health policies and legislation (World Health Organization—Assessment Instrument for Mental Health Systems, 2007). Consequently, mental illness has become the third most common disability in the Philippines, wherein six million Filipinos live with depression and anxiety. Because of this, the country has the third highest rate of mental disorders in the Western Pacific (Martinez et al., 2020). Also, the Philippine World Health Organization (WHO) Special Initiative for Mental Health conducted in 2020 showed that ≥3.6 million Filipinos suffer from at least one kind of mental, neurological, or substance use disorder (Department of Health, 2020). Suicide rates are reported to be at 3.2 per 100,000 population with higher rates among males (4.3/100,000) than females (2.0/100,000). However, these numbers may be underreported because suicide cases may sometimes be misclassified as “undetermined deaths” (Lally et al., 2019; Martinez et al., 2020). The WHO estimated that 154 million Filipinos suffer from depression, 1 million from schizophrenia, and 15.3 million from substance use disorders, while 877,000 die due to suicide every year (Department of Health, 2018). Thus, mental disorders could greatly affect employment and levels of education, most especially in ages 25 to 52 years (Hakulinen et al., 2019). It was found in a study by Hakulinen et al. (2020) that individuals with a severe mental disorder had notably lower levels of employment before, and more especially after, the diagnosis of their disorder. Their overall incomes came primarily from transfer payments, and the most affected were those diagnosed with schizophrenia. After receiving a mental disorder diagnosis, more than half of these individuals received no employment earnings.

Filipinos are generally unhappy not only because of poor economic conditions (unemployment, low salary, etc.), but also because of pressures arising from high expectations from family and society. In the study by Palaganas et al. (2017), it was shown that 24% of midwives, 29% of doctors, 51% of nurses, and 61% of physical therapists desired to migrate to the United States, Canada, Australia, and the United Kingdom to work as health professionals. Their migrations resulted in shortages of health workers, reductions in the provision of health services, poor quality of health care service provision, longer waiting times for patients, and increased work overtime. Their decisions to migrate were mainly influenced by the greater number of employment opportunities and high salaries abroad where they are given more respect and quality of practice, which greatly develop their wellbeing.

**Figure 1:**
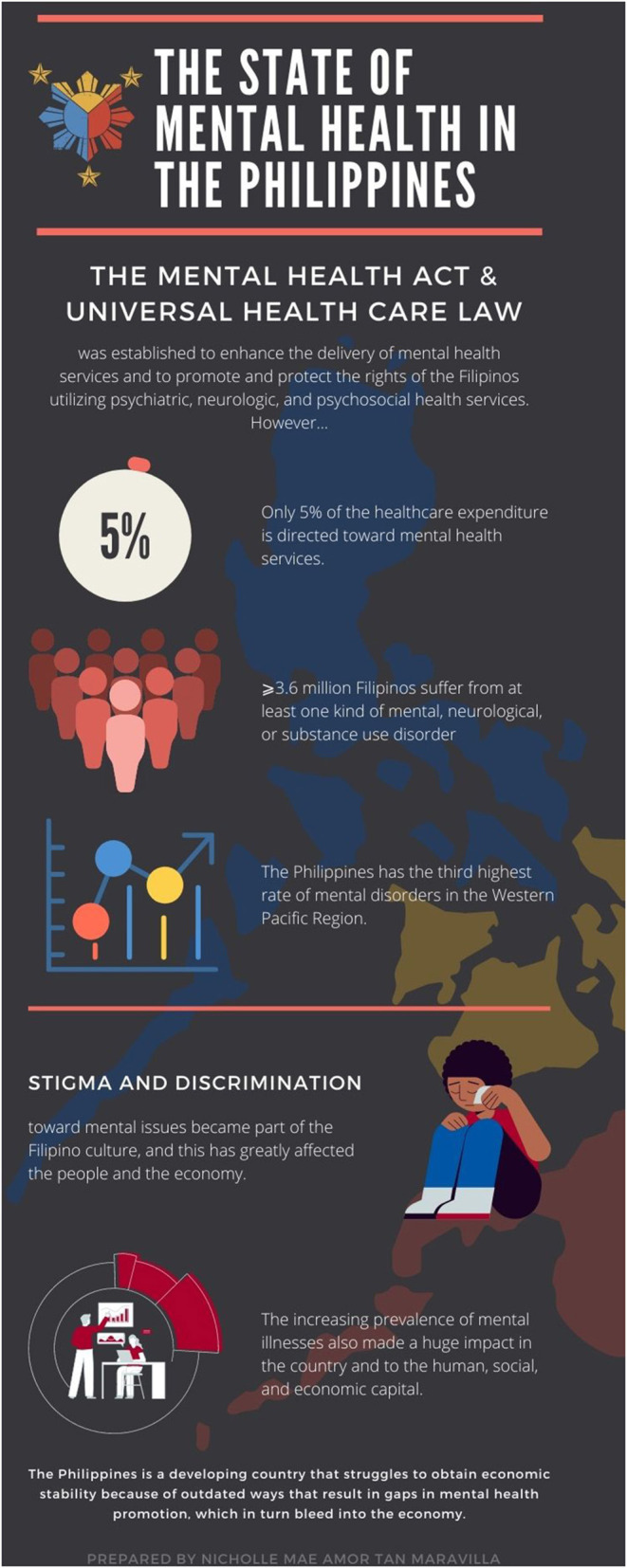
An infographic that summarizes the state of mental health in the Philippines.

The state of mental health in the Philippines in summarized in Figure 1 (World Health Organization—Assessment Instrument for Mental Health Systems, 2007; Department of Health, 2018; Tanaka et al., 2018; Martinez et al., 2020).

## A Happy Northwestern Europe

Here, we talk about the condition in Northwestern European countries to establish a basis with which to compare that in the Philippines.

Mental health is associated with either happiness or unhappiness, and is significant to Nordic life. A good reason why Nordics are happy is their system that provides security, free education, and a reasonable balance of work and personal time (Andreasson and Birkjaer, 2018). Humanitarianism is the root of their culture that has led to economic growth and reconversion of cities. Innovation and development were also attributed to their kindness toward the multicultural neighborhoods of their country (European Economic and Social European, 2016). Aside from culture, the government also plays an important role in developing these countries. Since the late 19th century, independent court systems that handle corruption-related matters have been distinctive features of Nordic legal systems. These have made their governments more trustworthy and reliable, and have given their citizens the assurance of effective reforms that fulfill their purpose (World Happiness Report, 2021). This explains why Northwestern European countries have among the highest scores in the World Governance Indicator. Moreover, they use digital tools to optimize management, service delivery, and overall state capacity. National websites are established to allow citizens' participation in policymaking and to provide feedback on public services. Thus, the collaboration between governments and their citizens is able to strengthen research, monitoring, and the evaluation framework of policymaking. It also promotes transparency and trust between them (World Health Organization, 2018b). In addition, health has always been their top priority. Therefore, public services such as healthcare and disability services are supported by the government (World Bank Group, 2021).

## The Philippines and Northwestern Europe—a Comparison

The World Bank Group (2021) has emphasized that mental health is part of the strategy to improve disadvantaged economies. In order to achieve this, people should be in positive mental and emotional states of enjoyment and contentment, *id est* “happiness” (Richards et al., 2015). However, according to Hart et al. (2018) and Wahlbeck (2015), socioeconomic factors like poverty, poor education, unemployment, and high debt, which are mostly experienced by Filipinos, affect happiness.

Economic crises can produce secondary mental health effects that lead to increased suicide and substance abuse mortalities (World Health Organization—European Region, 2020). The Philippines has faced multiple financial crises since the 1980s. In fact, the 1980s and 1990s saw a dramatic decline in the country's banking system that caused Filipinos and financial intermediaries to lose trust in it. This has been known as the *lost decade* for the whole Philippine economy. Fortunately, bank earnings recovered in the 2000s due to the economic, financial, and structural reforms that were implemented. This provided a strong economic foundation for the country, especially during the 2009 Global Financial Crisis. The country also continued to carry out policy reforms that further enhanced the various sectors of the economy. However, severe and shifting weather patterns, and disease, have increasingly become a source of downside risks to the financial system (Bangko Sentral ng Pilipinas, 2020). In the study by Montagnoli and Montagnoli (2019), it was shown that financial crises bring about micro- and macroeconomic changes in countries that affect SWB and lead to major and long-lasting psychological losses. However, these losses can be counteracted by social welfare and other policy measures (World Health Organization—European Region, 2020). Thus, measures of the same nature should be carried out to counteract the effects of financial crises in the Philippines and in turn, alleviate the plight of low-wage earners who are more prone to psychological distress (Kronenberg et al., 2017). These measures can be achieved by investing a greater deal in health and education.

However, Filipinos migrate to other countries because of the persistent economic crises and unemployment. Palaganas et al. (2017) showed that the decision of the workers to migrate is mainly influenced by their job dissatisfaction with income, workload, and infrastructure. However, increasing the wages of low-wage earners would not entirely enhance their mental health (Kronenberg et al., 2017). According to Martinez et al. (2020), although Filipinos enjoy more opportunities and higher wages abroad, mental health issues still exist among Overseas Filipino Workers due to language barriers, immigration status, lack of insurance, and discrimination. This may be explained by the happiness-income paradox presented by Easterlin et al. (2010). According to them, “at a point in time both among and within nations, happiness varies directly with income, but over time, happiness does not increase when a country's income increases.” This is evidence of the incompleteness of the unidirectional perspective of the effects of the economy on happiness, and in turn, mental health. While there is truth in this perspective, the perspective in the other direction, i.e. that the effect of mental health on the economy, is probably equally as important.

Being that mental health issues have been widely neglected in the Philippines and migration has resulted in the loss of investments in human capital (Palaganas et al., 2017), it would be best to take steps to attempt to adopt the mental health promotion strategy of Europe as nine of the 10 happiest countries in the world—Finland, Denmark, Switzerland, Iceland, Netherlands, Norway, Sweden, Germany, and Austria are in Northwestern Europe (World Happiness Report, 2021). This is perhaps because of their good economic status and quality healthcare. Mental health promotion in these countries proved to be more effective because it was introduced into the political agenda using a different approach. Neither the prevalence of mental illness nor the need for resources was emphasized. Instead, mental health was highlighted as a fundamental component of public health, and as having a significant effect on individual countries and their human, social, and economic capital (Wahlbeck, 2015). If the Department of Health in the Philippines would use this approach, the government would view the bottomline of the economic crisis in a different light. Realizing this would provide a new perspective to managing the economy.

However, the unresponsive governance has made Filipino people of all socio-demographic profiles distrust the government. This leads to a disunity between the two groups. According to Montagnoli (2019), uncertainty and distrust in financial and political institutions caused by financial crises may result in psychological losses that shape decision making. These crises exacerbate disapproval and distrust in the Philippine government. This is contrary to the experiences in Northwestern Europe, where individuals trust and socially interact with their neighborhoods. Finland, for instance, which ranked highest on measures of mutual trust, enjoys the sentiment that their lives and livelihoods are protected even during times of the pandemic (Hart et al., 2018; World Happiness Report, 2021).

As regards psychiatric services provided in Northwestern European countries, there are 39 to 130 psychiatric beds per 100,000 inhabitants and over 80,000 psychiatrists. Of these countries, those with the highest numbers of psychiatrists were Germany (27 per 100,000 inhabitants), Finland (24 per 100,000), the Netherlands (23 per 100,000), and Sweden (23 per 100,000) (Eurostat, 2020). However, suicide and mental disorders still exist in these countries. In 2015, it was reported that there were 56,000 deaths due to suicide in the European Union. And, among Northwestern European countries, Belgium recorded the highest suicide rate (17 per 100,000 population) while Demark recorded the lowest (10 per 100,000) (Eurostat, 2018). The prevalence of mental disorders are also very high in Europe, where Finland (18,800 per 100,000) and the Netherlands (18,600 per 100,000) ranked the highest in the continent. In all European countries, especially in Northwestern Europe, the most common mental health problem is anxiety disorder (25 million people), followed by depressive disorders (21 million), alcohol and drug use disorders (11 million), bipolar disorders (5 million), and schizophrenia (1.5 million) (OECD/European Union, 2018). It certainly appears that mental health problems are significantly more prevalent in Northwestern European countries than in the Philippines. However, this is probably because mental disorders go underdiagnosed in the Philippines owing to lack of mental health providers and facilities in the country.

Moreover, the economic and social burden of mental illnesses, unemployment, and worker productivity losses amount to over 600 billion Euros or 4% of the gross domestic product (GDP) across 28 European countries. The government has already spent around three-quarters of its health funds, and the country's national budget may be affected if large healthcare spending continues. There would also be challenges on the fiscal sustainability of health and long-term care systems. For these reasons, health sectors have made major strides to promote good mental health and to prevent mental illnesses. Moreover, many more European countries have ensured the implementation of comprehensive plans and policies that address mental health promotion and suicide prevention. The European Mental Health Action Plan 2013-2020 is strong evidence of this (OECD/European Union, 2018) and the fruits of these efforts are manifested by their favorable SWB scores (De Neve and Sachs, 2020) and by the findings of the World Happiness Report (2021). The architects of the mental health program of the WHO in Europe, its member states, and their partners worked together to develop and implement mental health policies and legislations that reflect the vision of the WHO that there is “no health without mental health” (World Health Organization—European Region, 2018). If the Philippines were also to endeavor toward this sense of solidarity, there, too, would be improvements in the healthcare system that would send ripples throughout the economy.

Although there were years of slow health economic growth across Europe following the economic crisis in 2008, nearly all European countries were able to rise in recent years. Yet, there are variations observed in the level and growth of health spending across Europe. For instance, high-income European countries, such as Luxembourg, Norway, and Switzerland, have the highest health expenditures per capita at EUR 4,713 (approximately USD 5715 in today's exchange rates), while Romania (EUR 983 or USD 1,192) and Bulgaria (EUR 1,234 or USD 1,496) have the lowest (OECD/European Union, 2018). Nevertheless, the mental health expenditure per capita in Europe is higher than all other countries at EUR 17.89 (USD 21.70). Also, 77% of the countries in Europe have stand-alone mental health laws, while 64% of them have updated these legislations since 2013. Meanwhile, in the Philippines,mental health and other economic problems are hardly addressed because of undeveloped mental health legislations, plans and policies (World Health Organization, 2018a), and annual net fiscal loss (Department of Finance, 2021). Even though the country's Current Health Expenditure (CHE) reached 792.6 billion (USD 16.5 billion) in 2019, 10.9% higher than 714.8 billion (USD 14.9 billion) in 2018, the total mental health expenditure per person is only 12.19 (USD 0.25). This is only 1/87 that of Europe. Moreover, only 0.22% of the government's total expenditure is allotted to mental health. The bulk of the CHE was spent on hospitals (43.6%), followed by pharmacies (30.3%), and providers of health care system administration and financing (7.4%). There are also stand-alone laws and policies for mental health in the country but there are no reports that monitor their implementation. Neither are there authorities that assess the compliance of mental health legislations in the Philippines with international human rights (World Health Organization—Assessment Instrument for Mental Health Systems, 2007; Philippine Statistics Authority, 2020). Evidently, mental health economists are needed to best resolve the mental health issues of the country.

Mental health is not well-established in the Philippines because of the dearth of investments channeled toward research. Thus, Filipino mental health workers cannot fully utilize their skills due to outdated practice guidelines and inappropriate curricula (Palaganas et al., 2017) unlike in Europe where guidance on economic crises and mental health are based on carefully reviewed research (Carrasco et al., 2016). The implementation of community mental health services are also based on empirical clinical evidence. Through this, they are able to recognize gaps that exist between the needs of the population and actual service provision (Semrau et al., 2011). This commitment has led to technological innovations such as e-Mental Health after evaluating the efficacy of delivering mental health services (Gaebel et al., 2020).

## Discussion

Thus far, the Philippine Mental Health Act has been nothing more than “just an act.” Nonetheless, there is still hope that the provision of mental healthcare will be recognized as a significant need to ameliorate life and economy. However, gradual change should begin with norms ingrained in culture before governmental reforms could be enjoyed, as these, too, are products of social norms themselves. Moreover, social stigma and discrimination are the toxic traits that misshape Filipino culture.

Furthermore, this is the time to drop the romanticization of Filipino resiliency because the truth is that the average Filipino is not genuinely happy. A picture of a smiling Filipino does not equate to a happy Filipino because the mentally ill know how to smile too. The Philippines is a developing country that struggles to obtain economic stability because of outdated ways that result in gaps in mental health promotion, which in turn, bleed into the economy. The increasing prevalence of mental illnesses bears a great impact on human, social, and economic capital. This may be true not only in the Philippines, but also in other developing countries. Moreover, depression and anxiety should be recognized as disorders, not mere illusions. Families must be listeners and comforters of the mentally ill, not castigators. Filipinos must also understand that there is a complex process in managing mental health issues and full recovery could not be achieved over a short period of time.

This article is a call for Filipinos to view mental health issues in a different light and to impel government and public sectors to prioritize them and to set the Philippine Mental Health Act into motion. Below is a prescription for the realization of a mentally healthy Philippines.

First, mental health professionals must be mobilized to educate families about mental health and mental disorders to eliminate stigma and discrimination. They must participate in and contribute to the development of mental health policy and service delivery guidelines. And very importantly, “family group conferencing” skills should be included in the training and practice of psychiatry.

Second, since mental disorders usually begin in adolescence, much attention on the mental health of individuals in this age group must be given. Suicide intervention, prevention, and response strategies with particular attention to the concerns of the youth should be implemented.

Third, the quality of mental health services should be based on the findings of medical and scientific research. By doing so, a comprehensive and effective mental health care system could be developed and established to provide the psychological, psychosocial, and neurologic needs of the Filipino. Family members should also be encouraged to participate in research, in formulating and developing mental health policies, and in promoting mental health in the workplace and communities.

And finally, because suicide and substance abuse continue to be prevalent in the country, it would be best for legislators to review the Mental Health Act in order to identify any lapses in the law for its improvement.

Through these efforts, we hope that the Philippine Mental Health Act would be able to effectuate happiness, contentment, and healthier social relationships. These will be good not only for the mental health of the individual, but also for those around him or her. The mentally healthy Filipino population that emerges through these changes could reverse the effects of financial crises, unresponsive governance and unproductivity in the country. The economy will continue to grow, employment and salaries will increase, and Filipinos will no longer need to migrate abroad to seek greener pastures.

As earlier mentioned, MHE is a subject that cries out for exploration in the Philippines. Alas, here, mental health and the economy are considered to be two separate concepts that appear to exclude each other. Be that as it may, we hope that this article sparks conversations that will draw the indubitable connection between these correlated concepts in academe, government, and industry. We hope that it becomes a discipline in itself and investigations in the discipline are carried out for the good of the country. We hope that this paper would interest future researchers to look into verifying the converse of the seemingly intuitive idea that a healthy economy makes people happy as it is our belief that happy people will make our economy healthy.

## Author Contributions

Conceived the work: MT and NM. Drafted the article: NM. Critically revised the manuscript: MT. Both authors read and approved the final manuscript.

## Conflict of Interest

The authors declare that the research was conducted in the absence of any commercial or financial relationships that could be construed as a potential conflict of interest.
